# Potential role of ATM in hepatocyte endocytosis of ApoE-deficient, ApoB48-containing lipoprotein in ApoE-deficient mice

**DOI:** 10.3892/ijmm.2013.1566

**Published:** 2013-11-26

**Authors:** JIANHUA WU, YANHONG XIAO, JUANG LIU, HONG YANG, XIAOMIN DONG, SAN HU, SHANRUI JIN, DONGFANG WU

**Affiliations:** 1 Department of Pharmacy, Zhongnan Hospital of Wuhan University, Wuhan, Hubei 430071, P.R. China; 2 Department of Osteology, Zhongnan Hospital of Wuhan University, Wuhan, Hubei 430071, P.R. China; 3 Department of Cardiovascular Biology, Meharry Medical College, Nashville, TN 37208, USA

**Keywords:** ataxia telangiectasia mutated, class III phosphatidylinositol-3-kinases, lipoprotein, hepatocytes

## Abstract

Individuals carrying mutations at both ataxia telangiectasia mutated (*ATM*) gene alleles reportedly have increased plasma cholesterol and triglyceride levels. Previous studies have demonstrated that defective ATM function promotes atherosclerosis. We previously demonstrated that ATM facilitates the clearance of plasma apolipoprotein (Apo)E-deficient, ApoB48-containing (E^−^/B^48^) lipoproteins in ApoE-deficient mice (*ApoE^−/−^* mice). However, to date there is no exact explanation available as to the mechanism(s) through which ATM is involved in the removal of E^−^/B^48^ lipoprotein in *ApoE^−/−^* mice. In this study, to our knowledge, we demonstrate for the first time that heterozygous *ATM* mutation reduces the hepatocyte uptake of E^−^/B^48^ lipoproteins in *ApoE^−/−^* mice; however, heterozygous *ATM* mutation did not affect hepatocyte binding to E^−^/B^48^ lipoproteins. Moreover, our results revealed that ATM proteins were localized in the nucleus, early endosomes and late endosomes, but not in the plasma membrane in the hepatocytes of *ApoE^−/−^* mice. In addition, following treatment with the ATM activator, chloroquine, and E^−^/B^48^ lipoproteins, ATM interacted with class III phosphatidylinositol-3-kinases (PI3Ks) and the activated ATM protein enhanced class III PI3K activity. Furthermore, treatment with a class III PI3K inhibitor (LY290042 and 3-MA) attenuated the intracellular total cholesterol accumulation induced by ATM activation. These results provide insight into the mechanisms behind the involvment of ATM in the process of endocytosis of E^−^/B^48^ lipoprotein in *ApoE^−/−^* mice, demonstrating the role of class III PI3K protein.

## Introduction

Patients with ataxia telangiectasia (A-T), carrying mutations at both ataxia telangiectasia mutated (*ATM*) alleles (*ATM^−/−^*), present with progressive cerebellar ataxia, susceptibility to cancer, immunodeficiency, insulin resistance and hyperglycemia (1,2). In addition, patients with A-T reportedly have increased plasma cholesterol and triglyceride levels (3), which are the two major risk factors for atherosclerosis. It is currently known that humans with the heterozygous *ATM* mutation (*ATM^+/−^*), which account for 0.5–2% of the total population, also have an increase risk of developing atherosclerosis-related cardiovascular diseases (4). Previous studies have suggested that defective ATM function promotes atherosclerosis through multiple systemic pro-atherosclerotic features, such as metabolic syndrome, oxidative stress, DNA damage and mitochondrial dysfunction (5,6). We have prevoiusly demonstrated that ATM assists the clearance of plasma apolipoprotein (Apo)E-deficient, ApoB48-containing (E^−^/B^48^) lipoproteins in ApoE-deficient mice (*ApoE^−/−^* mice) (7). However, to date, there is no exact explanation available as to the mechanisms through which ATM is involved in the endocytosis or removal of E^−^/B^48^ lipoproteins in *ApoE^−/−^* mice.

It is now known that a fraction of the ATM protein is also present in the cytoplasm and is associated with vesicular structures, such as peroxisomes, lysosomes and endosomes (8–10), which indicates that ATM may be involved in the trafficking of proteins and vesicles. Certain studies have reported that in the absence of ATM, intracellular vesicle and/or protein transport may be impaired, leading to abnormalities in endosomal function (11). Therefore, we hypothesized that *ATM* mutations in *ApoE^−/−^* mice may lead to an overaccumulation of plasma ApoB-48-containing lipoproteins, thus promoting the development of atherosclerosis, which may be associated with the loss of or the impaired function of cytosolic ATM protein. This may affect the process of endocytosis of lipoproteins or it may influence lipid metabolism enzymes involved in the endocytic scavenging process, and transport of proteins to the peroxisomes and/or lysosomes.

Phosphatidylinositol-3-kinases (PI3Ks), are known to play a key role in a wide range of cellular functions, including cell growth, proliferation, differentiation, motility and survival (12). It is also evident that PI3Ks play an important role in endocytosis and vesicle transport, including a role in the recruitment of regulatory proteins to the plasma membrane, endocytic uptake and recycling of receptors (13). Class III PI3Ks are responsible for the production of phosphatidylinositol-3-phosphate [PtdIns(3)P] (14), which is enriched in the membranes of early endosomes and the internal vesicles of multivesicular bodies (15,16). PtdIns(3)P recruits proteins containing FYVE, PX or PH motifs, and is involved in the control of vesicular transport and intracellular protein sorting (17,18). Certain studies have reported that PI3Ks are involved in the metabolism of different lipoproteins. For example, Shetty *et al* (19) demonstrated that PI3K plays an important role in class B type I scavenger receptor subcellular localization and selective lipid uptake in hepatocytes. Kzhyshkowska *et al* (20) reported that PI3K activity is required for the transfer of stabilin-1 and its ligand, acetylated low-density lipoprotein, from early endosomes to late endosomes. As ATM has been shown to possess a carboxyl-terminal domain homologous to PI3Ks and that the ATM protein regulates PI3K protein activity (21,22), we hypothesized that PI3K may involved in the promotion of E^−^/B^48^ lipoprotein endocytosis by cytoplasmic ATM in the hepatocytes of *ApoE^−/−^* mice.

In this study, we demonstrate that heterozygous *ATM* mutation reduces the hepatocyte uptake of E^−^/B^48^ lipoproteins in *ApoE^−/−^* mice and that the ATM protein is distributed in early and late endosomes. In addition, we reveal that the activated ATM protein interacts with class III PI3K and affects its activity and that a class III PI3K inhibitor attenuates the intracellular total cholesterol accumulation induced by ATM activation. The data presented in this study, provide insight into the mechanisms behind the involvement of ATM in the process of endocytosis of E^−^/B^48^ lipoproteins.

## Materials and methods

### Animals

*ATM^+/−^* mice were kindly provided by Dr Anthony Wynshaw-Boris (University of California, San Diego, CA, USA). *ApoB^48/48^/ApoE^−/−^* and 129vEv wild-type mice were obtained from the Jackson Laboratory (Bar Harbor, ME, USA). *ApoB^48/48^/ApoE^−/−^* mice were obtained by crossbreeding *ApoE*^−^*^/^*^−^ mice with *ApoB^48/48^* mice. *ATM^+/−^*/*ApoE^−/−^* mice were obtained by crossbreeding *ATM^+/−^* and *ApoE^−/−^* mice. In the present study, *ATM^+/−^*/*ApoE^−/−^*mice were used at 14 weeks of age. The mice appeared as healthy as their *ATM^+/+^*/*ApoE^−/−^* littermates. All procedures for handling the animals were approved by the Institutional Animal Care and Use Committees of Meharry Medical College (Nashville, TN, USA) and were performed in accordance with the guidelines of the American Association for the Accreditation of Laboratory Animal Care and the National Institutes of Health and Animal Care Guidelines of the Animal Experimental Committee of the College of Medicine, Wuhan University, Wuhan, China.

### Hepatocyte isolation and culture

Hepatocytes were isolated from *ApoE^−/−^* mice. After the mice were anesthetized and the livers exposed, the livers were first perfused with calcium-free buffer for 1.5 min and then perfused with 0.25% (w/w) collagenase type I (nitrogen) at 37°C in Williams E nutrient medium for 4 min. Subsequently, the hepatocytes were isolated by gently mincing the livers in Williams E nutrient medium containing 0.25% collagenase type I, filtered through a nylon gauze, and centrifuged twice for 5 min at 50 × g at 4°C. The cell pellets consisted of pure hepatocytes, as confirmed under a light microscope, and the viability of the cells was 90% as determined by trypan blue exclusion assay.

### Preparation of E^−^/B^48^ lipoproteins

E^−^/B^48^ lipoproteins were prepared from the plasma of *ApoB^48/48^*/*ApoE^−/−^* mice as previously described in the study by Wu *et al* (7). Briefly, mouse plasma was overlaid with KBr gradient solution (d<1.006) and centrifuged at 120,000 rpm for 2 h with a Sorvall Discovery M150 ultracentrifuge (Thermo Scientific, Waltham, MA, USA). The E^−^/B^48^ lipoproteins were collected, dialyzed in phosphate-buffered saline (PBS) (pH 7.4) containing 10 mM EDTA for 48 h at 4°C, and filtered through a 0.45 μm filter. One milliliter of E^−^/ B^48^ lipoproteins at a concentration of 1 mg/ml was mixed with 0.2 ml of 1 M glycine buffer in 0.25 M NaOH (pH 10) and then mixed with a solution containing 7 μl of 100 mM iodine monochloride, 7 μl of 100 μCi/μl ^125^I, and 25 μl of 1 M glycine buffer in 0.25 M NaOH (pH 10). Subsequently, the reaction mixture was incubated at room temperature for 10 min and then applied to a 10 DG chromatography column (Bio-Rad Laboratories, Hercules, CA, USA) to remove free iodine. The ^125^I-labeled E^−^/B^48^ lipoproteins were eluted with PBS (pH 7.4) and dialyzed extensively against PBS (pH 7.4).

### Binding and uptake assays

For the uptake experiments, the cells were washed twice with 2 ml of serum-free medium, containing 0.2% BSA. The cells were then incubated with 1 ml of the same medium, containing various concentrations of labeled E^−^/B^48^ lipoproteins [very-low-density lipoprotein (VLDL)] for 2 h or containing 20 μg/ml labeled E^−^/B^48^ lipoproteins for 30, 60, 120 and 240 min. For the determination of surface bound proteins, the cells were pre-chilled on ice for 30 min before washing and incubating in medium containing E^−^/B^48^ lipoproteins; the cells were then incubated with 1 ml of the same medium, containing various concentrations of labeled E^−^/B^48^ lipoproteins for 2 h or containing 20 μg/ml labeled E^−^/B^48^ lipoproteins for 30, 60, 120 and 240 min. Following incubation with E^−^/B^48^ lipoproteins, the medium was removed and the cells were washed twice with 2 ml of ice-cold PBS containing 0.2% BSA followed by 2 more washes with 2 ml of ice-cold PBS. The cells were lysed by the addition of 1 ml of 0.5 M NaOH and lysate was collected for the measurement of protein (10 μl of aliquot) and radioactivity taken up by the cells.

### Endosomal fraction isolation

Mouse liver endosomal fractions were isolated as prevously described by Chen *et al* (23). Mouse livers were homogenized in 20% (w/v) homogenization buffer containing 0.25 M sucrose, 3 mM imidazole (pH 7.4), 1.7 nM antipain, 2 nM leupeptin and 1 mM phenylmethylsulfonyl fluoride. The homogenate was centrifuged at 460 × g for 10 min, the supernatant was saved and the pellet was rehomogenized and centrifuged as described above. The pooled supernatant was centrifuged at 24,000 × g for 10 min, and the resulting supernatant (S2) was then centrifuged at 100,000 × g for 90 min. The resulting microsomal pellet (P3) was suspended in homogenization buffer (1.0 g starting liver/2 ml homogenization buffer) using 10 strokes at 1,000 rpm. The resuspended P3 was then diluted with an equal volume of 2.0 M sucrose in 3 mM imidazole buffer. Three milliliters aliquots of the resulting 1.15 M sucrose fraction were successively overlaid with 1.0, 0.86, 0.6 and 0.25 M sucrose solutions, all buffered with 3 mM imidazole. Following centrifugation at 100,000 × g for 3.5 h, 3 distinct fractions of average density 1.06, 1.09 and 1.12 g/ml were obtained. The 1.06 and 1.09 fractions were pooled to yield the late endosomal fraction, and the 1.12 g/ml fraction contained early endosomes.

### Western blot analysis

Endosomal fractions were isolated and prepared as described above. Endosomal proteins were extracted by a dual precipitation procedure. First, endosomal fractions were suspended in 20% trichloroacetic acid (TCA) with 20 mM DTT (1 g of starting liver/2 ml 20% TCA). Second, the suspension was allowed to precipitate on ice for 2 h and centrifuged at 1000 × g for 10 min, and the pellet was then suspended in acetone with 20 mM DTT (1 g of starting liver/2 ml acetone). Proteins in the suspension were precipitated at −20°C for 4 h and centrifuged at 1,000 × g for 5 min. Residual acetone was removed by lyophilization. The protein pellet was solubilized in lysis buffer, sonicated at 100 W for 30 sec, and centrifuged at 25,000 × g for 1 h. Plasma membrane proteins were extracted using the Plasma Membrane Protein Extraction kit (Abcam, Cambridge, MA, USA). Nuclear and cytoplasmic proteins from the cells were extracted using the Nuclear-Cytosol Extraction kit (BioVision, Inc., Milpitas, USA). Protein expression was determined by western blot analysis. Briefly, equal amounts of protein were separated by 7.0 or 10% SDS-polyacrylamide gel electrophoresis (SDS-PAGE) and elcetrophoretically transferred onto nitrocellulose membranes. After being blocked with TBST containing 5% bovine serum albumin, the membranes were incubated with primary antibodies against ATM (Santa Cruz Biotechnology, Inc., Santa Cruz, CA, USA) and phosphorylated ATM (p-ATM) protein (Cell Signaling Technology, Danvers, MA, USA) at 4°C overnight, followed by incubation with horseradish peroxidase-conjugated secondary antibody for 1 h at room temperature. The immunostaining was visualized by enhanced chemiluminescence, and the results were normalized to β-actin expression.

### Co-immunoprecipitation

The hepatocytes were pre-treated with chloroquine (an ATM activator) for 1 h and then treated with 40 μg/ml lipoproteins for 8 h. Cytoplasmic proteins (500 μg) from the cells extracted using the Nuclear-Cytosol Extraction kit were used to perform immunoprecipitation assays according to the manufacturer’s instructions (Pierce, Rockford, IL, USA). Briefly, the cytoplasmic lysates were incubated with ATM or class III PI3K antibody (Santa Cruz Biotechnology, Inc.) for 2 h at room temperature followed by the addition of protein A/G-Sepharose beads and further overnight incubation at 4°C with gentle rocking. The immunoprecipitates were washed 3 times with lysis buffer. The samples were then subjected to SDS-PAGE and immunoblotting.

### Class III PI3K activity assay

Cytoplasmic proteins from cells were extracted using the Nuclear-Cytosol Extraction kit (BioVision). The proteins were immunoprecipitated with anti-class III PI3K antibody for 2 h at room temperature, and subsequently incubated with A Sepharose beads overnight at 4°C. The precipitates were then washed with lysis buffer. The immunoprecipitated proteins were incubated with PIP substrates *in vitro*, and the PI(3)P products were measured with the use of class III PI3K ELISA kit (Echelon Biosciences, Salt Lake City, UT, USA) according to the manufacturer’s instructions.

### Measurement of cellular cholesterol

Hepatocytes grown in 75-mm culture flasks were pre-treated with 5 μM chloroquine, 10 μmol/l KU55933 (an ATM-specific inhibitor), 10 μM LY290042 (a class III PI3K inhibitor) or 5 mM 3-MA (a class III PI3K inhibitor) for 2 h and then incubated with 40 μg/ml E^−^/B^48^ lipoproteins for 22 h followed by a 12-h equilibrium in lipoprotein-free medium. Quantitative measurement of intracellular total cholesterol (TC) *in vitro* was analyzed using the method described in the study by Gamble *et al* (24). In brief, the hepatocytes were collected and lipids were extracted by the addition of chloroform:methanol (2:1). The lipid phase was collected, dried, and then dissolved in isopropanol containing 10% Triton X-100. The concentrations of TC were measured by enzymatic assays and normalized to total cellular protein levels.

### Statistical analysis

The data are expressed as the means ± SEM. Comparisons among multiple groups were performed using one-way ANOVA or two-way ANOVA followed by the Student-Newman-Keuls or Dunnett’s test. Differences were considered significant at P<0.05.

## Results

### Uptake of E^−^/B^48^ lipoproteins

**Figure 1:**
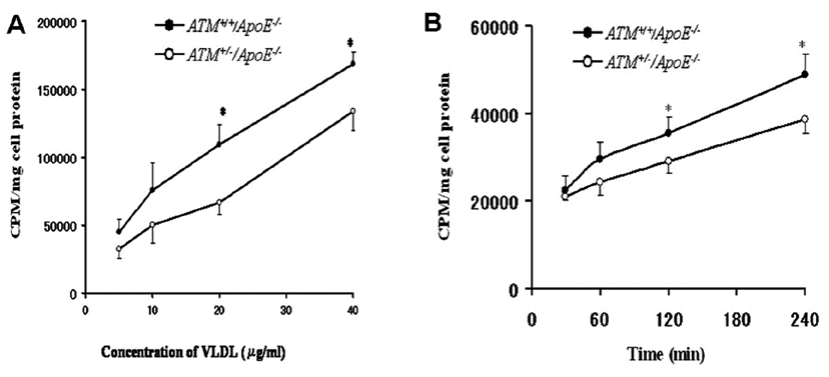
Uptake of ^125^I-labeled apolipoprotein (Apo)E-deficient, ApoB48-containing (E^−^/B^48^) lipoproteins by hepatocytes. Hepatocytes were isolated from ataxia telangiectasia mutated (*ATM)^+/−^*/*ApoE^−/−^* and *ATM^+/+^*/*ApoE^−/−^* mice by perfusion of the portal vein and incubated (A) with various concentrations of E^−^/B^48^ lipoproteins for 2 h and (B) with 20 μg/ml of E^−^/B^48^ lipoproteins for different periods of time at 37°C. The radioactivity in the cell lysates was measured as described in Materials and methods. Values represent the means ± SEM of 5 mice. ^*^Significant difference compared to *ATM^+/−^*/*ApoE^−/−^* mice (P<0.05). VLDL, very-low-density lipoprotein.

Both *ATM^+/−^*/*ApoE^−/−^* and *ATM^+/+^*/*ApoE^−/−^* hepatocytes absorbed E^−^/B^48^ lipoproteins in a concentration and time-dependent manner. However, the uptake of E^−^/B^48^ lipoproteins by the *ATM^+/+^*/*ApoE^−/−^* hepatocytes was greater than that of the *ATM^+/−^*/*ApoE^−/−^* hepatocytes. The uptake of E^−^/B^48^ lipoproteins by the *ATM^+/+^*/*ApoE^−/−^* hepatocytes was greater by 38–65% compared with the *ATM^+/−^*/*ApoE^−/−^* hepatocytes at the concentration range of 5–40 μg/ml; in particular, a significant difference was observed between the *ATM^+/−^*/*ApoE^−/−^* and *ATM^+/+^*/ *ApoE^−/−^* hepatocytes at the concentration range of 20–40 μg/ml and at the 120 and 240 min time points (P<0.05) (Fig. 1).

### Binding of E^−^/B^48^ lipoproteins

**Figure 2:**
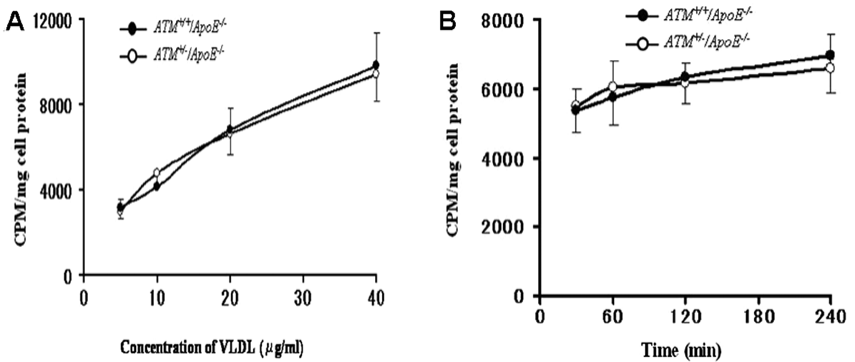
Binding of ^125^I-labeled apolipoprotein (Apo)E-deficient, ApoB48-containing (E^−^/B^48^) lipoproteins to hepatocytes. Hepatocytes were isolated from ataxia telangiectasia mutated (*ATM)^+/−^*/*ApoE^−/−^* and *ATM^+/+^*/*ApoE^−/−^* mice by perfusion of the portal vein and incubated (A) with various concentrations of E^−^/B^48^ lipoproteins for 2 h and (B) with 20 μg/ml of E^−^/B^48^ lipoproteins for different periods of time at 4°C. The radioactivity in the cell lysates was measured as described in Materials and methods. Values represent the means ± SEM of 5 mice. VLDL, very-low-density lipoprotein.

Both *ATM^+/−^*/*ApoE^−/−^* and *ATM^+/+^*/*ApoE^−/−^* hepatocytes bound E^−^/B^48^ lipoproteins in a concentration-dependent manner. However, no significant difference was observed in the binding of E^−^/B^48^ lipoproteins to the *ATM^+/−^*/*ApoE^−/−^* and *ATM^+/+^*/*ApoE^−/−^* hepatocytes at the concentration range of 5–40 μg/ml (Fig. 2A). The binding of E^−^/B^48^ lipoproteins to the *ATM^+/−^*/*ApoE^−/−^* and *ATM^+/+^*/*ApoE^−/−^* hepatocytes was not enhanced and no significant difference was observed as time progressed (Fig. 2B).

### Distribution of ATM protein in endosomes and ATM activation by chloroquine in the nucleus and cytoplasm

**Figure 3:**
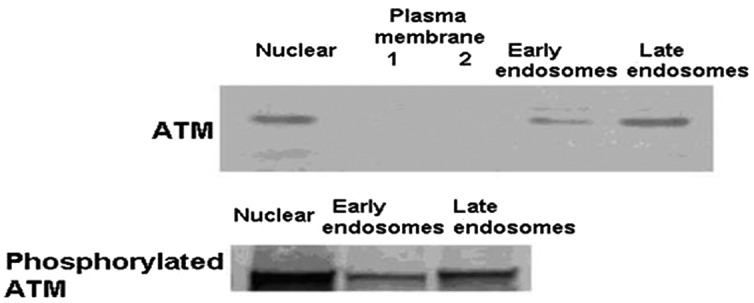
Ataxia telangiectasia mutated (ATM) and phosphorylated ATM protein in early and late endosomes in apolipoprotein (Apo)E-deficient (*ApoE^−/−^)* mice. Early endosomes (d=1.12 g/ml), late endosomes (d=1.06–1.09 g/ml) and cell nuclei were isolated by density ultracentrifugation. Plasma membrane proteins were extracted using the Plasma Membrane Protein Extraction kit. Nuclear proteins were extracted using the Nuclear-Cytosol Extraction kit. ATM and phosphorylated ATM protein expression was measured by western blot analysis as described in Materials and methods (n=3).

**Figure 4:**
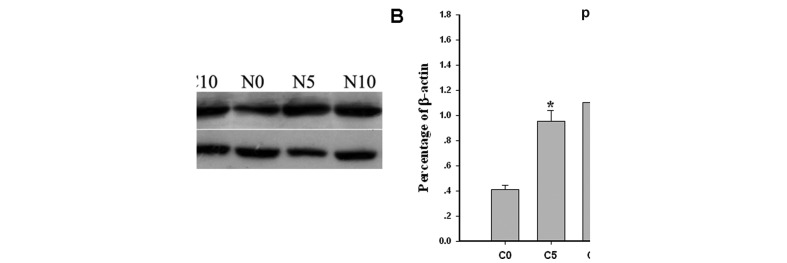
Ataxia telangiectasia mutated (ATM) activation by chloroquine in the nucleus and cytoplasm. (A) Representative protein bands from western blot analysis for phosphorylated ATM (p-ATM) and β-actin. (B) The intensity of the p-ATM band was quantified and the ratio of p-ATM to β-actin was calculated. Hepatocytes were isolated from apolipoprotein (Apo)E-deficient (*ApoE^−/−^*) mice by perfusion of the portal vein and incubated with various concentrations of chloroquine (0, 5 and 10 μmol/l) for 8 h at 37°C. ‘C’ indicates that the protein was from the cytoplasm. ‘N’ indicates that the protein was from the nucleus; 0, untreated group; 5, 5 μmol/l cholrquine-treated group; 10, 10 μmol/l chloroquine-treated group. Results are expressed as the means ± SEM (n=5). ^#^P<0.05 vs. N0 group; ^*^P<0.05 vs. C0 group.

ATM protein and p-ATM protein were expressed in the nucleus, early endosomes and late endosomes, but not in the plasma membrane in the hepatocytes of *ApoE^−/−^* mice (Fig. 3). The hepatocytes of *ApoE^−/−^* mice were incubated with various concentrations of chloroquine (0, 5 and 10 μmol/l) for 8 h at 37°C. p-ATM levels in the nucleus and cytoplasm increased following treatment with chloroquine in a dose-dependent manner (P<0.05) (Fig. 4).

### ATM protein interaction with class III PI3K protein and its effect on class III PI3K activity

**Figure 5:**
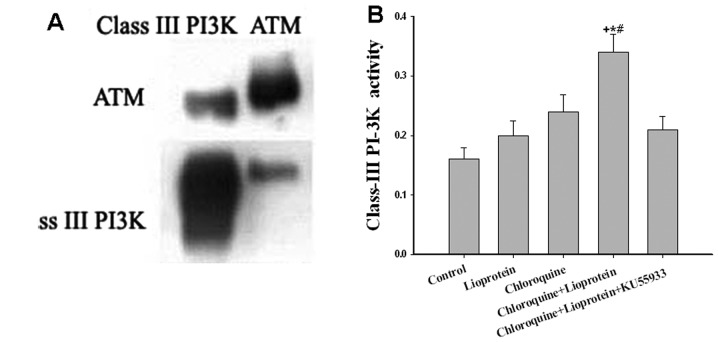
Analysis of the interaction betweetn ataxia telangiectasia mutated (ATM) and class III phosphatidylinositol-3-kinases (PI3Ks) by co-immunoprecipitation and augmentation of class III PI3K activity induced by the activation of ATM protein by chloroquine. (A) Representative protein bands from co-immunoprecipitation analysis between and ATM and class III PI3K protein. Hepatocytes from apolipoprotein (Apo)E-deficient mice (*ApoE^−/−^* mice) were pre-treated with 5 μmol/l chloroquine for 1 h and then treated with 40 μg/ml ApoE-deficient, ApoB48-containing (E^−^/B^48^) lipoproteins for 8 h at 37°C. Cells were collected and homogenated. Protein in the cytoplasm was extracted and immunoprecipitated with ATM and class III PI3K proteins. Western blot analysis was performed to determine whether the immunoprecipitates contained ATM and class III PI3K proteins. (B) Class III PI3K activity was assessed by ELISA. Hepatocytes from *ApoE^−/−^* mice were incubated with 5 μmol/l chloroquine, 40 μg/ml E^−^/B^48^ lipoproteins and 10 μmol/l KU55933 (an ATM inhibitor) for 8 h at 37°C. Cells were collected and centrifuged. Cytoplasmic proteins from the cells were extracted using the Nuclear-Cytosol Extraction kit. Class III PI3K protein in the cytoplasm was immunoprecipitated, and then its activity was determined using the class III PI3K activity ELISA kit. Values represent the means ± SEM of 5 mice. ^*^P<0.05 vs. control; ^#^P<0.05 vs. lipoprotein; ^+^P<0.05 vs. chloroquine + lipoprotein + KU55933.

As ATM has been shown to possess a carboxyl-terminal domain homologous to PI3Ks and that ATM protein regulate PI3K activitys, we wished to determine whether ATM interacts with class III PI3Ks and whether ATM activation affects the activity of class III PI3Ks. The results from co-immunoprecipitation analysis indicated that cytoplasmic ATM protein interacted with cytoplasmic class III PI3K protein (Fig. 5A). In addition, when the hepatocytes were incubated with E^−^/B^48^ lipoproteins alone, class III PI3K protein activity increased; however, no significant difference was observed between the control group (untreated group) and the group treated with E^−^/B^48^ lipoproteins (P>0.05). When the hepatocytes incubated with E^−^/B^48^ lipoproteins and chloroquine, class III PI3K activity markedly increased (P<0.01); however, this effect was attenuated by the ATM inhibitor, KU55933 (P<0.05) (Fig. 5B).

### Class III PI3K inhibitor attenuates intracellular total cholesterol accumulation induced by ATM activation

**Figure 6:**
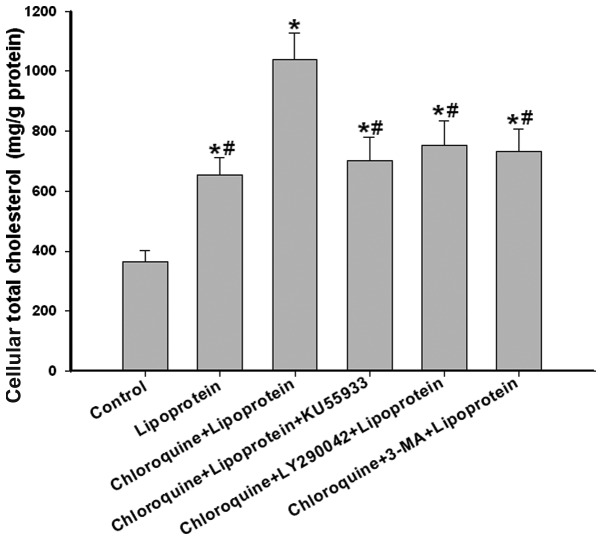
Class III phosphatidylinositol-3-kinase (PI3K) inhibitor attenuates intracellular total cholesterol accumulation induced by ataxia telangiectasia mutated (ATM) activation. Hepatocytes were isolated from apolipoprotein (Apo)E-deficient mice (*ApoE^−/−^* mice) by perfusion of the portal vein and pre-treated with 5 μM chloroquine, 10 μmol/l KU55933 (an ATM inhibitor), 10 μM LY290042 (a class III PI3K inhibitor) or 5 mM 3-MA (a class III PI3K inhibitor) for 2 h and then incubated with 40 μg/ml ApoE-deficient, ApoB48-containing (E^−^/B^48^) lipoproteins for 22 h at 37°C. Cellular total cholesterol in hepatocytes was determined using an enzymatic kit. Values represent the means ± SEM of 8 mice. ^*^P<0.05 vs. control; ^#^P<0.05 vs. choroquine + lipoprotein.

When the hepatocytes were incubated with E^−^/B^48^ lipoproteins alone, the intracellular total cholesterol of the hepatocytes increased, compared with the control (untreated) group (653.8±58.2 vs. 362.5 ± 38.2 mg/g protein). When the hepatocytes were incubated with E^−^/B^48^ lipoproteins and chloroquine, the intracellular total cholesterol of the hepatocytes markedly increased compared with the lipoprotein-treated group (1038.5±88.3 vs. 653.8±58.2 mg/g protein). However, treatment with the ATM inhibitor, KU55933, and the class III PI3K inhibitor, LY290042 and 3-MA, abolished the intracellular total cholesterol accumulation induced by chloroquine (702.8±78.6 vs. 1038.5±88.3 mg/g protein), (753.3±82.5 vs. 1038.5±88.3 mg/g protein) or (732.2±76.3 vs. 1038.5±88.3 mg/g protein), respectively (Fig. 6).

## Discussion

The major findings of the present study are as follows: i) the uptake of E^−^/B^48^ lipoproteins by the *ATM^+/+^*/*ApoE^−/−^* hepatocytes was greater than that of the *ATM^+/−^*/*ApoE^−/−^* hepatocytes, although no significant difference was observed in the binding of E^−^/B^48^ lipoproteins between the *ATM^+/+^*/*ApoE^−/−^* and *ATM^+/−^*/*ApoE^−/−^* hepatocytes; ii) a fraction of the ATM protein was expressed in early endosomes and late endosomes, but not in the plasma membrane; iii) ATM protein interacted with class III PI3K protein and the activated ATM protein enhanced class III PI3K activity; iv) the class III PI3K inhibitor, LY290042, abolished the intracellular total cholesterol accumulation induced by ATM activation.

Under physiological conditions, ApoE activates receptor-mediated endocytosis by binding to cell surface low-density lipoprotein (LDL) receptor and LDL receptor-related protein (LRP) (25). The deletion of ApoE, lipoproteins containing ApoB100 (such as LDL) can still be achieved by the interaction between ApoB100 and LDL receptor, but lipoproteins containing ApoB48 can not enter cells through the LDL receptor and LRP (26). We previously reported that *ATM* heterozygous mutation in *ApoE^−/−^* mice resulted in an overaccumulation of plasma ApoB48-containing lipoproteins and severe hypercholesterolemia occurred only with a combination of a heterozygous *ATM* mutation and a null *ApoE* mutation, but not wih the heterozygous *ATM* mutation alone or with the combined heterozygous *ATM* and null *LDL* receptor mutations (7). The present experimental results revealed that despite the lack of ApoE, lipoproteins containing ApoB48 can still be absorbed. Compared with hepatocytes of *ATM^+/+^*/*ApoE^−/−^* mice, the uptake of E^−^/B^48^ lipoproteins by hepatocytes of *ATM^+/−^*/*ApoE^−/−^* mice decreased significantly; however, no signficant difference was observed in the binding of E^−^/B^48^ lipoproteins between hepatocytes from *ATM^+/+^*/*ApoE^−/−^* mice and those of *ATM^+/−^*/*ApoE^−/−^* mice. Based on these results, it can be concluded that there are other pathways mediating the endocytosis of lipoproteins containing ApoB48 without ApoE, and ATM facilitates the endocytosis of lipoproteins containing ApoB48. In addition, we found that a portion of the ATM protein was localized in early endosomes and late endosomes, but not in the plasma membrane. These results indicate that the ATM protein may participate in the endocytosis of E^−^/B^48^ lipoproteins.

Since we observed that the ATM protein was expressed in endosomes and it is known that class III PI3Ks are responsible for the production of PtdIns(3)P in the membranes of endosomes, and mediate vesicular transport, membrane trafficking and intracellular protein sorting (14,27,28), our study focused on the effects of ATM on the activity of class III PI3Ks. Certain studies have reported that small doses of chloroquine activate nucleic ATM proteins (29). In this study, we observed that chloroquine activated ATM in the nucleus and cytoplasm of hepatocytes in a dose-dependent manner. ATM has been shown to possess a carboxyl-terminal domain homologous to PI3Ks and ATM protein has been shown to interact with PI3K, regulating PI3K activity (21,22). Similar to this result, in our present study, we observed an interaction between cytoplasmic ATM and class III PI3Ks; activated ATM increased class III PI3K protein activity. Moreover, chloroquine, which activated ATM protein, promoted intracellular total cholesterol accumulation, while the class III PI3K inhibitor, LY290042 and 3-MA, inhibited this effect, suggesting that class III PI3K protein plays an important role in the ATM protein-mediated endocytosis of E^−^/B^48^ lipoproteins in the hepatocytes of *ApoE^−/−^* mice.

Previous studies have confirmed that there are ApoE-independent mechanisms which mediate the uptake of lipoproteins containing ApoB48. Magoori *et al* (30) reported that *ApoE* and *LRP-5* double knockout mice developed more severe hypercholesterolemia than *ApoE^−/−^* mice and that this hypercholesterolemia resulted mainly from an increased level of ApoB-48-containing lipoproteins in the plasma, wheras *LRP-5* single knockout mice showed no significant difference in plasma cholesterol levels. These results indicate that the LRP-5 mediates the ApoE-independent plasma lipoprotein metabolism pathway. Therefore, it is possible that E^−^/B^48^ lipoproteins interact with cell membrane receptors, such as LRP-5 through unknown mechanisms, and may activate cytosolic ATM; activated ATM in turn activates class III PI3K, regulating the endocytosis of E^−^/B^48^ lipoproteins. This may facilitate E^−^/B^48^ lipoprotein degradation and metabolism. Based on these results, we hypothesized that the ATM/class III PI3K pathway may be involved in the endocytosis of E^−^/B^48^ lipoproteins. In addition, it is evident that chloroquine exerts an inhibitory effect on blood lipids; however, the exact mechanisms involved are unclear. Our results suggest that the decrease in blood lipid levels by chloroquine may be associated with the activation of the ATM/class III PI3K pathway, thus promoting the uptake of lipoproteins by hepatocytes.

In conclusion, to the best of our knowledge, the results presented in this study demonstrate for the first time that a heterozygous mutation in *ATM* reduces the uptake of E^−^/B^48^ lipoproteins by hepatocytes of *ApoE^−/−^* mice. We also found that ATM was distributed in early endosomes and late endosomes. Our results demonstrated that the ATM protein interacted with class III PI3K protein and the activated ATM protein enhanced class III PI3K activity. In addition, the ATM activation promoted intracellular total cholesterol accumulation; however, this was abolished by the class III PI3K inhibitor, LY290042. These observations suggest that ATM is involved in the endocytosis of E^−^/B^48^ lipoproteins through the class III PI3K protein. Our findings provide further insight into the mechanisms through which the ATM activation by chloroquine exerts beneficial effects, reducing blood lipid levels.
